# Assessment of Liver Remnant Using ICG Clearance Intraoperatively during Vascular Exclusion: Early Experience with the ALIIVE Technique

**DOI:** 10.1155/2015/757052

**Published:** 2015-05-27

**Authors:** Lawrence Lau, Christopher Christophi, Mehrdad Nikfarjam, Graham Starkey, Mark Goodwin, Laurence Weinberg, Loretta Ho, Vijayaragavan Muralidharan

**Affiliations:** ^1^Department of Surgery, Austin Health, University of Melbourne, Melbourne, VIC 3084, Australia; ^2^Department of Radiology, Austin Health, Melbourne, VIC 3084, Australia; ^3^Department of Anaesthesia, Austin Health, University of Melbourne, Melbourne, VIC 3084, Australia

## Abstract

*Background*. The most significant risk following major hepatectomy is postoperative liver insufficiency. Current preoperative assessment of the future liver remnant relies upon assumptions which may not be valid in the setting of advanced resection strategies. This paper reports the feasibility of the *ALIIVE* technique which assesses the liver remnant with ICG clearance intraoperatively during vascular exclusion. *Methods*. 10 patients undergoing planned major liver resection (hemihepatectomy or greater) were recruited. Routine preoperative assessment included CT and standardized volumetry. ICG clearance was measured noninvasively using a finger spectrophotometer at various time points including following parenchymal transection during inflow and outflow occlusion before vascular division, the ALIIVE step. *Results*. There were one case of mortality and three cases of posthepatectomy liver failure. The patient who died had the lowest ALIIVE ICG clearance (7.1%/min versus 14.4 ± 4.9). Routine preoperative CT and standardized volumetry did not predict outcome. *Discussion/Conclusion*. The novel ALIIVE technique is feasible and assesses actual future liver remnant function before the point of no return during major hepatectomy. This technique may be useful as a check step to offer a margin of safety to prevent posthepatectomy liver failure and death. Further confirmatory studies are required to determine a safety cutoff level.

## 1. Introduction

Surgical resection remains the foundation for curative treatment of liver malignancies. Resection strategies balance the goal of macroscopic tumour clearance and preserving adequate future liver remnant (FLR) [1]. Inadequate FLR leads to posthepatectomy liver failure (PHLF) as defined by deterioration in the ability of the liver to maintain its synthetic, excretory, and detoxifying functions [2]. This is the most common cause of mortality following hepatectomy [3]. Current assessments of FLR are based on computed tomography (CT) imaging and are contingent upon the predicted volume of liver remnant either as a percentage of the total preoperative liver volume (CT volumetry) or as a percentage of an ideal total liver volume as calculated by body surface area (standardized volumetry) [4]. Increasingly, advanced strategies, which enhance technical resectability, have gained prominence. These strategies include preoperative chemotherapy [5], combining local ablation with resection [6], portal vein embolisation [7, 8], 2-staged resection [9], and recently the associating liver partition and portal vein ligation for staged hepatectomy (ALPPS) technique [10]. While rapid liver hypertrophy induced by these advanced techniques has increased resectability, increase in parenchymal volume has not been shown to definitively correlate with increased functional liver capacity. In the climate of these advances, the applicability of current assessment is uncertain. The presence of patient-related factors such as age, diabetes, and obesity and parenchyma-related factors such as cirrhosis, cholestasis, steatosis, and chemotherapy injury further cloud idealized volumetry [11–14]. Functional FLR measurement using hepatobiliary scintigraphy has been shown to be a better predictor in those with parenchymal disease [15, 16]. However, both techniques suffer due to potential discrepancy between the planned and actual transection planes. Furthermore, the functional contribution of liver parenchyma that is poorly perfused or has poor venous drainage after transection is impossible to predict [17–19].

Indocyanine green (ICG) is tricarbocyanine dye taken up exclusively by hepatocytes and excreted into bile without enterohepatic recirculation [20, 21]. It is widely used to evaluate preoperative liver functional reserve [22–26] and as an early indicator of outcome following liver resection [22, 23, 25, 27] and orthotopic liver transplantation [28–34].

In this report, we describe a novel technique which may be used intraoperatively to assess true FLR. This study assesses the technical feasibility and early experience of the novel ALIIVE technique in the assessment of liver remnant using ICG clearance intraoperatively during vascular exclusion of the liver being resected. This technique may potentially be utilized as a final safety check step that evaluates the sufficiency of the actual future remnant before the irreversible step of vascular division. Potentially, if insufficient future liver remnant function was found at this step, the planned hepatectomy may be converted to an ALPPS procedure. Conversely, in the setting of a planned ALPPS procedure, if sufficient future liver remnant was confirmed during the ALIIVE check step, completion of the hepatectomy as a single-staged procedure may prevent reoperation and the morbidity associated with a two-staged procedure.

## 2. Methods

This prospective technical feasibility study was approved by Austin Health Human Research Ethics Committee (project number: HREC/13/Austin/150). Signed, written informed consent was obtained from each patient.

### 2.1. Patient Selection

From February 2014 to August 2014, consecutive patients planned for hemihepatectomy or greater at Austin Health in Melbourne, Australia, were recruited for this study. All patients were discussed at the Austin Health Hepatobiliary Multi-Disciplinary Team Meeting prior to planned resection.

### 2.2. Routine Future Liver Remnant Assessment

Routine preoperative FLR assessment was performed using two techniques.

#### 2.2.1. CT Volumetry

Preoperative multiphase contrast-enhanced CT scans of the abdomen were obtained routinely as part of staging and planning for surgery with portal venous phase images reconstructed at 3 mm slice thickness. CT volumetry expresses the predicted FLR volume as a percentage of total liver volume based on the reconstructed images (%FLRV). Liver volumes were then calculated by a specialist hepatobiliary radiologist using VitreaWorkstation (Toshiba Medical, Tokyo, Japan) by manually drawing regions of interest around the areas of the liver to denote the volumes of the tumour (TV), FLR (FLRV), and total liver volume (TLV). Segments were demarcated according to the conventional Couinaud classification. The %FLRV is calculated by the following formula:

(1) $$\begin{array}{c} \%FLRV=\frac{FLRV}{TLV-TV}\times 100\%. \end{array}$$

#### 2.2.2. Standardized Volumetry

Similar to CT volumetry, reconstructed CT scans were used to calculate FLRV while standardized total liver volume (sTLV) was calculated based on patient body surface area (BSA) according to the following formula:

(2) $$\begin{array}{c} sTLV=-794.41+1267.28\times BSA, \end{array}$$

where

(3) $$\begin{array}{c} BSA=\sqrt{\frac{height\left(cm\right)\times weight\left(kg\right)}{3600}}. \end{array}$$

Standardized future liver remnant (sFLR) is calculated as

(4) $$\begin{array}{c} sFLR=\frac{FLRV}{sTLV}. \end{array}$$

A %FLRV or sFLR >20% and >30% in patients with normal and suspected diseased liver parenchyma (cholestasis, steatosis, and >6 cycles of preoperative chemotherapy) was considered sufficient [35–37].

### 2.3. ICG Clearance

**Figure 1:**
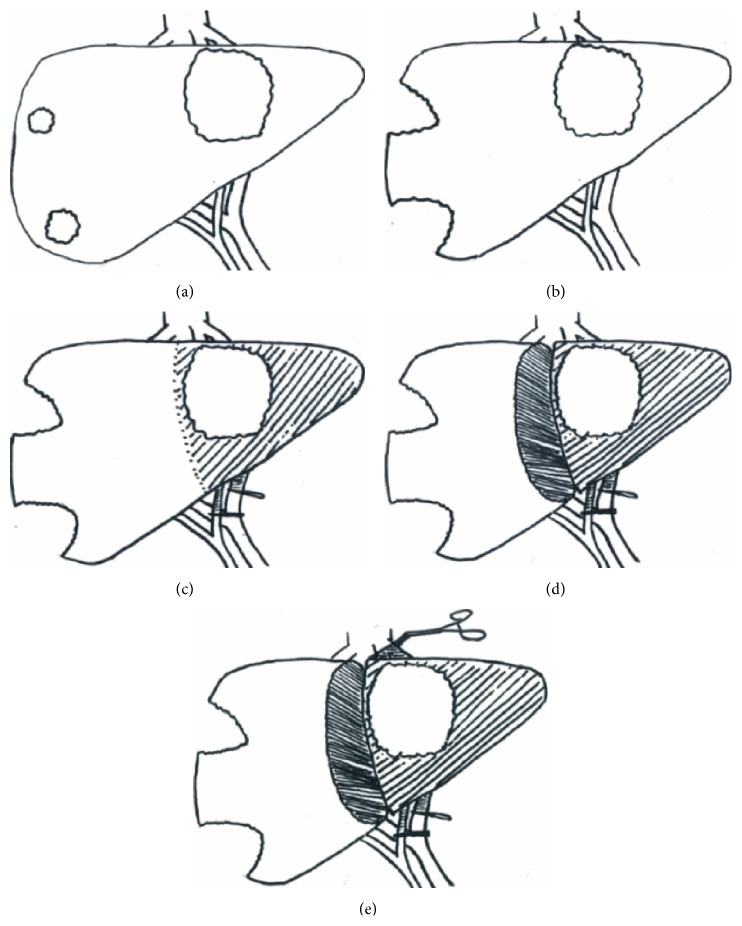
Schematic diagram of ICG clearance time points in a liver with a large left-sided tumour and two small superficial right-sided tumours: (a) ICG1: preoperative, (b) ICG2: under anaesthesia following clearance of future liver remnant, (c) ICG3: during inflow control to the side to be resected, (d) ICG4: during inflow control following parenchymal transection, and (e) ICG5: the ALIIVE step, during inflow and outflow control following parenchymal transection.

ICG clearance was assessed using the LiMON module of the PulsioFlex monitor (Pulsion Medical Systems, Munich, Germany) to obtain the PDR (%/min). ICG clearance was performed during the following time points (Figure 1): (1) before anaesthesia (ICG1), (2) under anaesthesia following laparotomy ± subsegmental tumour clearance of FLR (ICG2), (3) during inflow occlusion (hepatic artery and portal vein) to the lobe for resection (ICG3), (4) following parenchymal transection and inflow occlusion (ICG4), and (5) during inflow and outflow occlusion following parenchymal transection of the lobe for resection (ICG5 a.k.a. the ALIIVE step). For each ICG measurement, a bolus of 25 mg of ICG was injected into a central venous catheter. The ICG elimination was detected by noninvasive pulse spectrophotometry and the ICG PDR was automatically calculated within six minutes. A delay of 30 minutes was required between measurements. Maximum allowable daily dose is 5 mg/kg.

### 2.4. Anaesthesia

General anaesthesia was managed by specialist anaesthetists using a protocol designed to standardise care. Prior to induction of anaesthesia all patients received intrathecal morphine (300 *μ*g) inserted at the L3/4 intervertebral space. Induction of anaesthesia consisted of a balanced technique using propofol (1–3 mg/kg), fentanyl (1–3 *μ*g/kg), and a nondepolarising neuromuscular blocker. Maintenance of anaesthesia was achieved with sevoflurane or desflurane in a 50% oxygen : 50% air ratio titrated to a bispectral index (BIS) of 40 to 60. Intraoperative analgesia consisted of a remifentanil infusion (0.1 to 0.3 *μ*g/kg/min) that was discontinued prior to surgical closure. Intraoperative monitoring consisted of continuous electrocardiography, pulse oximetry, invasive blood pressure, central venous pressure, urine output, and core body temperature. Flow based haemodynamic variables (cardiac index, stroke volume variation, and stroke volume) were evaluated continuously with the PulsioFlex monitor using a ProAQT sensor (Pulsion Medical Systems, Munich, Germany). During the hepatic parenchymal resection phase, fluid therapy was limited and an infusion of glyceryl trinitrate (GTN) was used where necessary to achieve a central venous pressure of less than 5 mmHg. After hepatic resection, the GTN was discontinued and patients were rendered euvolemic. Vasoactive drugs (e.g., phenylephrine, noradrenaline, and metaraminol) were used to maintain blood pressure within 20% of the preoperative value. Fluid intervention was confined to Plasmalyte solution (Baxter Healthcare, Toongabbie, NSW, Australia). The intraoperative transfusion trigger for packed red cell transfusion was a haemoglobin concentration of less than 70 g/L or less than 80 g/L in the setting of ongoing or uncontrolled bleeding, myocardial ischaemia, or a low cardiac output state.

### 2.5. Surgical Resection

Intraoperative ultrasound was used for intraoperative intrahepatic staging, to identify tumour margin and hepatic anatomy. If required, subsegmental resection was performed to clear the future liver remnant of tumour. Anatomical resection was performed in accordance with Couinaud's liver segmental classification. Portal and hepatic arterial inflow to the segments for resection were dissected extrahepatically and occluded with vascular bulldog clamps to allow for ICG clearance assessment (ICG3). Liver parenchymal transection was performed using a combination of an ultrasonic surgical aspirator (CUSA, Radionics, Burlington, MA, USA), Harmonic shears (Ethicon, Somerville, NJ, USA), and linear surgical stapler (EndoGIA, Covidien, Mansfield, MA, USA; Echelon Endopath, Ethicon, Somerville, NJ, USA). Following complete parenchymal transection, an ICG clearance was performed with the inflow occluded and with outflow (hepatic vein) patent (ICG4). A final ICG clearance was performed with both inflow and outflow occlusion (ICG5). Following this step, the liver resection was completed with ligation and division of the hepatic artery, portal vein, and hepatic vein to the pertinent hemiliver.

### 2.6. Study Endpoints

Although this feasibility study was not designed to assess endpoints, postoperative liver failure and death due to postoperative liver insufficiency were assessed. All postoperative morbidities were recorded and graded according to the Clavien-Dindo classification [38]. Postoperative liver failure was defined according to the International Study Group of Liver Surgery (ISGLS) grading [2].

### 2.7. Statistics

Values for results are expressed as median with the range and mean ± standard deviation.

### 2.8. Results

In total, 10 patients participated in this feasibility study. Demographic and tumour information are listed in Table 1. The majority of patients underwent preoperative chemotherapy (7) and were trisegmentectomies (6) while half had preoperative portal vein embolization (5). In patients who underwent preoperative portal vein embolization, the median kinetic growth rate of the future remnant was 7.4%/week.

There were 3 cases of posthepatectomy liver failure, one of which was grade C liver failure. This patient died on the fifth postoperative day. Stratified by outcome (Table 1), the patients with posthepatectomy liver failure were older with cholangiocarcinoma.

#### 2.8.1. CT Volumetry

The median estimated liver remnant on CT volumetry was 46% (range 26–84%). The estimated remnant in the patient who died was 38%. For the other two patients with posthepatectomy liver failure, the estimated liver remnant was 45% and 46%.

#### 2.8.2. Standardized Volumetry

The median estimated liver remnant on standardized volumetry was 57%. This was 49% in the patient who died. In the patients with reversible liver failure, their standardized volumetry was 65% and 59%.

#### 2.8.3. Intraoperative ICG Clearance

**Figure 2:**
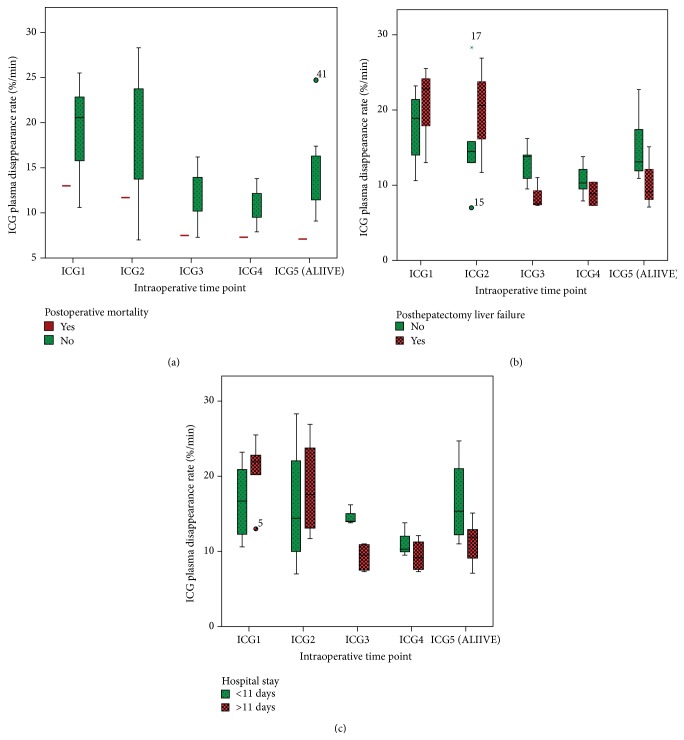
Interquartile box-plots of ICG clearance at different time points stratified by (a) postoperative mortality, (b) posthepatectomy liver failure, and (c) hospital stay.

The intraoperative ICG clearances at the various time points are shown in Table 1. The patient who died had a lower ICG clearance at the ICG3, ICG4, and ICG5 time points (7.5, 7.2, and 7.1%/min) compared to the other patients (11.8 ± 3, 10.7 ± 2.1, and 14.4 ± 4.9%/min). The interquartile ranges stratified for outcome are displayed in Figure 2. Cardiac index was measured at each ICG time point and ranged from 1.9 to 3.6 (Table 1).

### 2.9. Discussion

This study describes a novel technique to assess FLR intraoperatively. In our early experience with these first ten cases, the technique was shown to be feasible and its potential for use as a safety check step was demonstrated along with alternate treatment strategies in the setting of apparent inadequacy of the FLR.

ICG clearance is a functional liver test which has been in established use for planning of surgical resection and for monitoring patients following liver resection for liver insufficiency [22–27]. Following intravenous injection, ICG is taken up by hepatocytes and excreted with bile via an ATP-dependent mechanism. As hepatic ATP is important for liver viability, regeneration, and metabolic function, ICG clearance correlates with global liver function [39].

This study assessed the use of ICG clearance intraoperatively at various time points during liver resection. Time points ICG3, ICG4, and ICG5 demonstrate increasing degrees of vascular exclusion of the FLR. Interestingly, increasing vascular exclusion led to decreased ICG clearance in some cases but, in other cases, it led to improved clearance. One explanation for this observation relates to haemodynamic variability during the different time points. Another reason is the potential for interlobar crossover flow [40] in ICG3 or retrograde hepatic vein flow [41] in both ICG3 and ICG4. This could either lead to better ICG clearance by the functional contribution of the additional parenchyma or to decreased ICG clearance due to ICG stasis in the nonexcluded but diseased parenchyma. Therefore, the ALIIVE step, which essentially replicates the hepatectomized state, should best predict the outcome.

This pilot study was not intended, nor was it powered, to determine a safety cutoff level for ICG clearance during vascular exclusion. However, an indication of a safety cutoff level can be extrapolated from the previous studies assessing ICG clearance postoperatively [22, 23, 25, 27]. In a study by Sugimoto et al., following liver resection, a PDR <7%/min on postoperative day 1 was found to be highly predictive for liver insufficiency (sensitivity 71.4%, specificity 95.5%) and death (sensitivity 100%, specificity 93.6%) [27]. In another study by Ohwada et al., patients who had liver failure following hepatectomy had a median postoperative PDR of 7.6%/min [23]. They proposed the use of an estimated remnant PDR cutoff (based on a product of CT volumetry and preoperative ICG clearance) of 9%/min to be 88% sensitive and 82% specific in the prediction of posthepatectomy liver insufficiency. In the study by de Liguori Carino et al. from Liverpool, ICG clearance was found to be uniquely useful for the early detection of posthepatectomy liver dysfunction where those with liver dysfunction had a significantly lower postoperative day 1 PDR compared to those who did not (6.75%/min versus 13.4%/min, *P* = 0.014) [22].

As the ALIIVE technique replicates the postresection state intraoperatively, it is reasonable to assume that a PDR that can be demonstrated to be greater than 9%/min would give a margin of safety while a PDR that cannot be demonstrated to be greater than 7%/min would be at high risk of liver insufficiency and death if hepatectomy was completed.

Correspondingly, in our small series, our only postoperative mortality had an ALIIVE ICG clearance of 7.1%/min prior to completion of hepatectomy, which is significantly lower than the other ICG clearances in patients at this time point who had their hepatectomy completed. This patient had a cholangiocarcinoma who underwent preoperative portal vein embolisation before resection. FLR grew from 431 mL (24%) to 710 mL (38%) and was deemed to be sufficient based on preoperative CT and standardized volumetry. Preoperative bilirubin was slightly elevated at 25 *μ*mol/L and histology revealed background liver parenchymal chronic cholestasis. This patient died following progressive liver failure likely due to small-for-size syndrome without sepsis or apparent surgical complications.

During this study, another patient had unexpected colorectal liver metastases on the FLR which were cleared. Following parenchymal transection, the decision was made (independent of ICG clearance) to convert the procedure to an ALPPS procedure. At this point, an ALIIVE ICG clearance was demonstrated to be 7.9%/min which, according to the studies of ICG clearance after hepatectomy, would be considered low. Two weeks later at the second stage hemihepatectomy, the ALIIVE ICG clearance had functionally increased to 11.9%/min and the liver resection was completed without complication (the ICG clearance from the first operation was not included in the overall analysis). Although management was not altered due to the ICG findings in this example, it displays the real-time intraoperative decision-making potential of the ALIIVE technique and demonstrates the functional increase of the actual future liver remnant following volume manipulation.

Following validation studies, the ALIIVE technique may potentially be used as a “check step” during major hepatectomy to avoid posthepatectomy liver failure. If there is insufficient FLR, possible alternative strategies may include conversion to a staged resection, ALPPS procedure with the addition of portal vein ligation, portal vein embolization, local ablative therapy, or palliative chemotherapy. Conversely, demonstration of sufficient FLR during a planned ALPPS procedure with the ALIIVE technique may allow confident hepatectomy and prevent unnecessary morbidity and subsequent reoperation. However, the potential benefit gained in preventing posthepatectomy liver failure will need to be balanced against the possible increase in technical difficulty with this technique as well as the potential complications in leaving the sectioned but unresected hemiliver in situ, in a prospective randomized control trial.

Although this technique would not replace preoperative volumetry, it has the potential to be a valuable adjunct to current assessment. The ALIIVE technique directly assesses FLR without making assumptions about the health of liver parenchyma, the actual resection plane, and the actual functional contribution of remnant liver parenchyma.

Liver parenchymal diseases such as nonalcoholic fatty liver disease, cirrhosis, biliary obstruction, and injury secondary to preoperative chemotherapy are associated with increased risk of liver insufficiency following liver resection. Both CT and standardized volumetry are based on the assumption of normal, homogenous liver parenchyma where a compensatory “guess” is applied with known parenchymal disease. Hepatobiliary scintigraphic functional volumetry does attempt to redress the issue of parenchymal disease but suffers the same deficiencies of being unable to predict the exact resection plane, consider the impact of impaired venous drainage, or adapt to altered intraoperative circumstances [15, 16]. The correlation of liver volume and function with more recent FLR growth techniques such as PVE and ALPPS is even less established.

A meta-analysis assessing the sensitivities of various imaging modalities following neoadjuvant chemotherapy found MRI and CT to have a pooled sensitivity of 85.7% and 69.9%, respectively, compared to intraoperative palpation with intraoperative ultrasound [42]. This means that around one out of every three to six liver tumours may be undetected on preoperative imaging requiring unplanned intraoperative resection. Additionally, the parenchymal transection plane may not follow a planned two-dimensional vertical axis as predicted by CT volumetry. Furthermore, for hemihepatectomies where the middle hepatic vein is excised the adjoining liver segments may have compromised venous drainage rendering them functionally impaired. The degree of impaired function is not taken into account with preoperative volumetric planning.

The main limitation of this technique relates to the contribution of vascular perfusion to the FLR ICG clearance. ICG clearance is a function of two processes: hepatic clearance and hepatic perfusion. The latter may be decreased by general anaesthesia, decreased cardiac output, decreased volume status, and hepatic artery vasospasm leading to impaired ICG clearance.

At each ICG clearance time point, cardiac index was measured and recorded to ensure that this was sufficient. If a low clearance was observed during the final vascular exclusion test, the vascular clamps were released and the clearance was repeated after 20 minutes with correction of any potential perfusion-limiting factors. These include increasing cardiac index with volume filling, allowing the liver to sit naturally without manipulation of the hepatic inflow, and spraying 5 mL of papaverine over the remnant liver hepatic artery to counteract potential vasospasm. Provided adequate vascular exclusion of the liver parenchyma to be resected, it is unlikely that ICG clearance assessment of the FLR can be falsely increased. The objective is to demonstrate the best possible FLR function, which, if adequate, should provide reassurance for safe resection. This technique requires the preservation of inflow and outflow until after parenchymal transection, which adds technical complexity to the operation.

This paper describes and reports the first use of the ALIIVE technique. This technique is feasible and early experience reveals it to be a compelling tool for intraoperative assessment and decision-making before irreversible vascular division to prevent posthepatectomy liver failure and death. This technique may be particularly pertinent in cases with diseased background liver parenchyma or where preoperative assessment suggests questionable future liver remnant sufficiency. The limit of safe ICG clearance during vascular exclusion remains to be confirmed by future validation studies.

## Figures and Tables

**Table 1:** Demographic, preoperative/operative factors and ICG clearance stratified by outcome.

Demographics	Median (% or range)	Dead	Alive	Posthepatectomy liver failure	No posthepatectomy liver failure
Number	10	1	9	3	7
Sex (male : female)	8 : 2	male	7 : 2	3 male	5 : 2
Age	60.9 (19–76)	75	63.7 ± 11.8	70.8 ± 4.5	62.5 ± 13.0
BMI	23.4 (21–30)	22.8	25 ± 3.2	25.2 ± 3.1	24.8 ± 3.3

*Preoperative factors *					
Colorectal metastases	6	0	6	0	6
Cholangiocarcinoma	4	1	3	3	1
Preoperative chemotherapy	7	0	7	1	6
Portal vein embolisation	5	1	4	2	3
If PVE-kinetic growth rate (%/week)	7.4 (6.47–9.07)	6.6	7.38 ± 1.2	7.0 ± 0.6	7.8 ± 1.8
Trisegmentectomy	6	1	5	1	5
Right hemihepatectomy	3	0	3	2	1
Left hemihepatectomy	1	0	1	0	1
Future liver remnant volume (mL)	760 (351–1221)	710	877 ± 299	869 ± 196	859 ± 328
CT volumetry (%)	46 (26–84)	38	53 ± 18	43.0 ± 4.4	54.5 ± 19.9
Standardized volumetry (%)	57 (22–66)	49	55 ± 17	57.9 ± 8.3	53.3 ± 19.0
Bilirubin (*μ*mol/L)	12 (5–35)	25	13 ± 10	10 ± 5.4	23 ± 13.1

*Operative factors *					
Blood loss (mL)	650 (200–1500)	1200	660 ± 568	700 ± 707	775 ± 585
Operating time (min)	510 (360–840)	540	518 ± 165	500 ± 69	534 ± 195

*ICG clearance *					
ICG 1: preoperative	21.4 (12.2–25.5)	14.2	19.2 ± 5.2	24.2 ± 1.9	17.8 ± 5.0
ICG 2: under anaesthesia ± clearance of future liver remnant	15.2 (7.0–28.3)	11.7	18.0 ± 7.7	19.7 ± 7.6	15.7 ± 7.8
ICG 3: inflow control	11.0 (7.3–16.2)	7.5	11.8 ± 3.0	8.6 ± 2.1	12.9 ± 2.7
ICG 4: inflow control following parenchymal transection	10.3 (7.8–13.8)	7.8	10.7 ± 2.1	10.4 ± 3.8	10.7 ± 2.3
ICG 5: inflow and outflow control following parenchymal transection (ALIIVE)	12.9 (7.1–24.7)	7.1	14.4 ± 4.9	10.4 ± 4.2	15.2 ± 5.1
					
Cardiac index (ICG2)	2.4 (2.1–3.3)	2.1	2.6 ± 0.5	2.1 ± 0.1	3.0 ± 0.4
Cardiac index (ICG3)	2.8 (1.9–3.2)	2.6	2.7 ± 0.5	2.3 ± 0.4	3.1 ± 0.2
Cardiac index (ICG4)	3.0 (2.3–3.2)	3.0	2.8 ± 0.5	2.7 ± 0.5	3.2 ± 0.5
Cardiac index (ICG5)	3.0 (2.4–3.6)	3.1	2.9 ± 0.5	2.8 ± 0.4	3.4 ± 0.5

*Outcome *					
Hospital stay (days)	11 days (5–48)	n/a	16 ± 14	33 ± 21	10 ± 4
Posthepatectomy liver failure	3 (30%)				
Grade A: abnormal lab parameters	1 (10%)				
Grade B: deviation from routine clinical management without invasive treatment	1 (10%)				
Grade C: deviation from routine clinical management requiring invasive treatment	1 (10%)				
